# Identification of Genes and Construction of Prognostic Model of Lung Adenocarcinoma Based on Propionate Metabolism-Related Genes

**DOI:** 10.14740/wjon2680

**Published:** 2026-01-04

**Authors:** Min Min Li, Wei Jia Fu, Ying Zhou, Huan Huan Zhang, Hai Ning Li, Chu Zhang, Jin Yang

**Affiliations:** a Department of Clinical Laboratory, The Affiliated Lianyungang Hospital of Xuzhou Medical University/The First People’s Hospital of Lianyungang, Lianyungang, Jiangsu 222000, China; b Department of Clinical Laboratory, The First People’s Hospital of Lianyungang, Lianyungang, Jiangsu 222000, China; c These authors contributed equally to this work.

**Keywords:** LUAD, Propionate metabolism, Hub genes, Prognostic model, Bioinformatics

## Abstract

**Background:**

Dysregulation of propionate metabolism can enhance the invasive properties of lung adenocarcinoma (LUAD) cells and increase their metastatic potential. Therefore, we constructed a predictive model based on propionate metabolism-related genes (PMRGs) to evaluate the prognosis of patients with LUAD.

**Methods:**

mRNA expression profiles and clinical data of LUAD patients were obtained from The Cancer Genome Atlas (TCGA) and Gene Expression Omnibus (GEO). The predictive model was constructed using least absolute shrinkage and selection operator (LASSO). The associations between the risk score and tumor prognosis, immune infiltration, drug sensitivity, signaling pathways, and clinical features were evaluated using the CIBERSORT algorithm, the Genomics of Drug Sensitivity in Cancer (GDSC) database, gene set variation analysis (GSVA), gene set enrichment analysis (GSEA), and nomogram analyses. For hub genes, motif enrichment, genome-wide association study (GWAS) analysis and single-cell analysis were performed. Expression differences between LUAD cells and normal lung epithelial cells were validated using quantitative real-time polymerase chain reaction (qRT-PCR).

**Results:**

We established a LUAD prognostic model containing 16 hub genes. The area under the receiver operating characteristic curve (AUC) values for the model were 0.722 (1-year overall survival (OS)), 0.696 (3-year OS), and 0.700 (5-year OS). GSVA revealed significant enrichment in the G2/M checkpoint, E2F targets, and glycolysis pathways. GSEA indicated enrichment of the hypoxia-inducible factor-1 (HIF-1), interleukin (IL)-17, and p53 signaling pathways. Transcription factor analysis identified the motif cisbp-M4287 as the most significantly enriched, with a normalized enrichment score (NES) of 5.9. qRT-PCR results showed that the expression of ADIPOQ, CYP27A1, and GCDH was downregulated, while the expression of SERPINE1 was upregulated. No statistically significant differences were observed in the expression of CYP17A1 and EHHADH.

**Conclusions:**

A novel prognostic model based on PMRGs was established to predict overall survival in LUAD patients.

## Introduction

Most cancer-related deaths are attributed to metastasis, a process characterized by progressive, heterogeneous, and systemic features [1]. Lung cancer is usually asymptomatic in the early stage or presents only with atypical early symptoms, resulting in most patients being diagnosed at stage III or IV, when the cancer cells have spread locally or metastasized [2]. Different types of lung cancer show distinct metastatic patterns and survival outcomes, highlighting their heterogeneity [3]. Lung adenocarcinoma (LUAD) is the most common non-small cell lung cancer (NSCLC), with the most common metastatic sites being brain and bone metastases, whereas small cell lung cancer (SCLC) predominantly spreads to the liver and brain [4]. Despite significant advances in the diagnosis and treatment of lung cancer, the prognosis remains poor with a low 5-year survival rate.

The alteration of metabolic pathways is one of the key characteristics of enhanced invasiveness of tumor cells [5]. Recent studies suggest that propionate plays an important role in cancer biology [6]. Studies have shown that dysregulation of propionate metabolism leads to the accumulation of malonic acid, which promotes cancer cell invasion and metastasis [7, 8]. A high-salt diet increases propionate, which promotes glioma invasion and progression through hypoxic response and transforming growth factor (TGF)-β signaling pathway [9]. Propionate treatment can also inhibit MEK/ERK phosphorylation in an Or51e2-dependent manner and promote apoptosis of mouse colorectal cancer cells [10]. Therefore, the role of propionate in tumor is complex. It can inhibit tumors by regulating immunity and inducing apoptosis, or promote tumor progression under certain conditions.

Epithelial-mesenchymal transition (EMT) is a key biological process in which tumor cells acquire invasive and metastatic capabilities [11]. Propionate is negatively correlated with EMT in LUAD [12]. Propionate can enhance epithelial cell properties, thereby inhibiting the invasiveness of lung cancer cells. This mechanism may involve propionate regulating epithelium-intermembrane-related gene expression and thereby inhibiting EMT [13]. Additionally, as a short chain fatty acid, propionate can enhance T-cell activity and improve the effect of immunotherapy for NSCLC [14]. Therefore, the construction of a prognostic model for LUAD based on propionate metabolism-related genes (PMRGs) is crucial for the development of new therapeutic strategies and improving patient survival.

In this study, we obtained the optimal risk score value for each sample through least absolute shrinkage and selection operator (LASSO) regression analysis. Multi-omics methods were used to explore the relationship between risk scores and tumor prognosis, immune infiltration, drug sensitivity, specific signaling pathways, and clinical indicators. Through genome-wide association study (GWAS) analysis and single cell analysis, we conducted comprehensive analyses of pathogenic regions in hub genes and their distribution across different cell subpopulations. Notably, our 16-gene PMRG-based model showed better prognostic performance than any single clinical variable and provided incremental prognostic value when combined with conventional clinical factors. The model has been validated across multiple datasets, showcasing remarkable predictive accuracy.

## Materials and Methods

### Data acquisition

**Figure 1:**
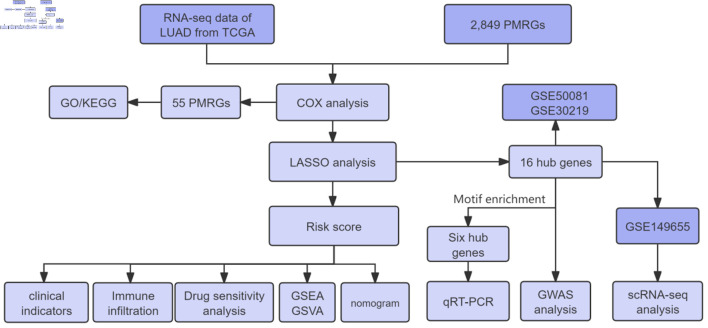
Overall workflow of this study. LUAD: lung adenocarcinoma; TCGA: The Cancer Genome Atlas; PMRGs: propionate metabolism-related genes; GSEA: gene set enrichment analysis; GSVA: gene set variation analysis; LASSO: least absolute shrinkage and selection operator; GO: Gene Ontology; KEGG: Kyoto Encyclopedia of Genes and Genomes; qRT-PCR: quantitative real-time polymerase chain reaction.

RNA sequencing (RNA-seq) data for LUAD were obtained from The Cancer Genome Atlas (TCGA) database [15], including normal groups (n = 59) and tumor groups (n = 541). A total of 2,849 PMRGs were retrieved from the GeneCards database. The LUAD microarray datasets GSE30219 and GSE50081 were obtained from the Gene Expression Omnibus (GEO) database [16]. Both datasets were annotated using the GPL570 platform. The GSE30219 cohort contains transcriptomic profiles and survival data for 85 patients, whereas GSE50081 includes similar data for 127 patients. Single-cell RNA-seq data (GSE149655) were also downloaded, providing scRNA-seq profiles from four LUAD patients for single-cell analysis. The workflow of this study is illustrated in Figure 1.

### Functional enrichment analysis of PMRGs in LUAD

We used the ClusterProfiler package in R to conduct functional enrichment analysis of the prognostic genes [17]. Gene Ontology (GO) [18] and Kyoto Encyclopedia of Genes and Genomes (KEGG) [19] analyses were conducted to identify enriched functional categories.

### Model construction and prognostic evaluation

Patients were randomly divided into a training set and a validation set at a ratio of 4:1. LASSO regression was performed using 10-fold cross-validation, and the optimal λ value was selected according to the minimum lambda criterion. After LASSO selection, the corresponding regression coefficients for the retained genes were obtained [20]. Based on the risk score formula, patients were divided into low-risk and high-risk groups using the median risk score as the cutoff point. The survival difference between the two groups was evaluated using the Kaplan-Meier method, and log-rank statistics were employed for comparison. Stratified analyses were conducted to further evaluate the prognostic value of the risk score. Receiver operating characteristic (ROC) curves were used to assess the accuracy of the model’s predictions.

### Immune cell infiltration analysis

CIBERSORT was applied to estimate the relative proportions of 22 immune-infiltrating cell types in low-risk and high-risk groups [21]. Pearson correlation analysis was performed to assess associations between gene expression and immune-cell proportions. P < 0.05 was considered statistically significant.

### Drug sensitivity analysis

Based on the one of the largest pharmacogenomic resources, the Genomics of Drug Sensitivity in Cancer (GDSC) database [22], the “pRRophetic” package in R was used to predict the chemotherapy sensitivity of each tumor sample [23]. The estimated half-maximal inhibitory concentration (IC_50_) values were compared between the high- and low-risk groups to identify drug candidates potentially influenced by the model-related target genes and to evaluate whether the predicted drug responses aligned with the biological mechanisms suggested by our model.

### Gene set variation analysis (GSVA) and gene set enrichment analysis (GSEA)

GSVA and GSEA were used to further analyze the differences in signaling pathways between low-risk and high-risk groups. Annotated gene sets were obtained from the MSigDB database [24] and used as reference collections for pathway enrichment analysis. Genes with significant enrichment (adjusted P < 0.05) are ranked based on their consistency scores.

### Nomogram model construction

A nomogram was developed using a multivariate Cox proportional hazards model that incorporated the LASSO-derived risk score together with clinical variables. Regression coefficients from the Cox model were converted into a point-based scoring system, with higher points indicating greater contribution to mortality risk. Each patient’s total score was calculated by summing the points of all predictors, and the corresponding 1-, 3-, and 5-year survival probabilities were estimated from the model’s baseline survival function. The predictive performance of the nomogram was assessed using ROC curves, calibration plots, and decision curve analysis (DCA) [25].

### Regulatory network analysis of hub genes

The “RcisTarget” package in R was used to predict transcription factors (TFs) regulatory motifs [26]. The rcistarget.hg19.motifdb.cisbpont.500bp was used as the gene-motif rankings database. In this analysis, a motif denotes a recurrent TF binding sequence enriched within the regulatory regions of the input gene set. The prefix “cisbp,” followed by a numerical code (e.g., cisbp_M4287), corresponds to a specific TF-binding motif indexed in the human Catalog of Inferred Sequence Binding Preferences (CIS-BP) database.

### GWAS analysis

The Gene Atlas database is a large database that utilizes the UK Biobank cohort to record associations between hundreds of traits and millions of genetic variants [27]. These associations were derived from data on 452,264 UK individuals in the UK Biobank, encompassing 778 phenotypes and 30 million loci in total.

### Single-cell analysis

#### Quality control (QC)

Single-cell expression profiles were processed using the Seurat package. Cells were filtered according to four quality metrics: total unique molecular identifier (UMI) counts, number of detected genes, percentage of mitochondrial reads, and percentage of ribosomal reads. Outliers were defined as measurements exceeding three median absolute deviations (MADs) from the median. Cells with abnormally high UMI or gene counts were flagged as potential doublets, whereas those with elevated mitochondrial or ribosomal content were considered low-quality or apoptotic. DoubletFinder (v2.0.4) was subsequently applied to remove doublets and complete QC for each sample.

#### Data standardization

After QC, data were normalized using NormalizeData, followed by cell cycle scoring with CellCycleScoring. Highly variable genes were identified using FindVariableFeatures. The data were then scaled with ScaleData, regressing out the effects of mitochondrial and ribosomal genes as well as cell cycle signals. Linear dimensionality reduction was performed using RunPCA, and batch effects were corrected with Harmony. Nonlinear dimensionality reduction was conducted using RunUMAP. Cell neighbors were identified using FindNeighbors, and clusters were generated with FindClusters. Cell types were annotated by integrating CellMarker-based manual curation with automated classification via SingleR.

#### Pseudotime trajectory analysis

To investigate dynamic transcriptional changes, pseudotime analysis was performed using Monocle. This approach leverages the asynchronous nature of single-cell transcriptional states to infer developmental trajectories, enabling characterization of transitional cell states, lineage relationships, and gene expression dynamics within heterogeneous LUAD cell populations

### Cultivation of LUAD cells

The A549 cell line was obtained from KGI Bio (catalog number: KEGG3215-1), and the Beas-2B cell line was obtained from FuHeng Bio (catalog number: FH0319). A549 cells were cultured in F-12K medium supplemented with 10% fetal bovine serum (FBS), while Beas-2B cells were maintained in DMEM medium containing 10% FBS. All cells were incubated at 37 °C in a humidified atmosphere with 5% CO_2_ (Thermo Scientific, China).

### RNA extraction and quantitative real-time polymerase chain reaction (qRT-PCR)

Total RNA was extracted using TRIzol reagent (KeyGEN, China). RNA concentration and purity were measured using a spectrophotometer, and samples were stored at -80 °C until cDNA synthesis, which was performed using the KeyGEN reverse transcription kit (Cat. No. KGA1317). Gene-specific primers were designed as listed here (Supplementary Material 1, wjon.elmerpub.com). qRT-PCR was conducted on an Applied Biosystems 7500 Real-Time PCR System using SYBR Green qPCR Master Mix (MedChemExpress, China).

### Statistical analysis

Survival curves were generated by Kaplan-Meier method and compared by log-rank. Cox proportional hazards model was used for multivariate analysis. All statistical analyses were conducted in R software (version 4.2), and P < 0.05 was considered statistically significant.

### Institutional Review Board (IRB) approval

This study did not involve any human participants or patient-derived specimens. The experimental component was conducted exclusively using commercially available, well-established cell lines for qRT-PCR validation. All other analyses were performed based on publicly accessible datasets. Therefore, in accordance with relevant ethical guidelines and institutional policies, this study does not constitute human subject research, and approval from the IRB was not required.

### Ethical compliance

All procedures involving human data in this study were conducted in compliance with the ethical standards of the institutional and national research committees and with the 1964 Helsinki Declaration and its later amendments or comparable ethical standards.

## Results

### Identification and functional enrichment of PMRGs in the LUAD cohort

**Figure 2:**
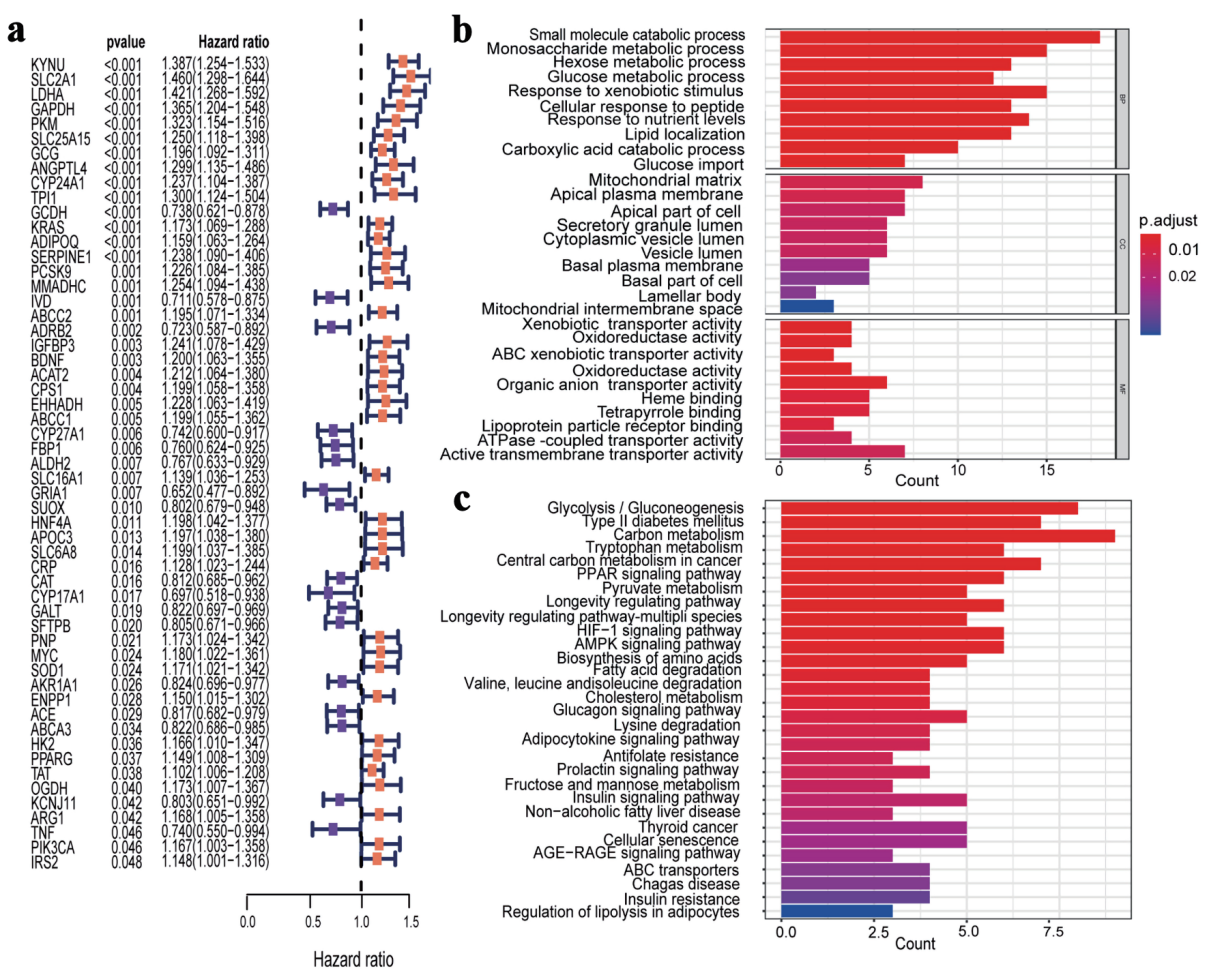
Identification and enrichment analysis of PMRGs in LUAD. (a) Cox univariate analysis selected 55 PMRGs. (b) GO analysis results for the 55 PMRGs. (c) KEGG analysis results for the 55 PMRGs. PMRGs: propionate metabolism-related genes; LUAD: lung adenocarcinoma; GO: Gene Ontology; KEGG: Kyoto Encyclopedia of Genes and Genomes.

A total of 340 PMRGs with a relevance score > 10 for LUAD from TCGA database were obtained. The Cox univariate regression results revealed 55 PMRGs associated with LUAD prognosis (P < 0.05) (Fig. 2a). GO enrichment analysis of the 55 PMRGs showed that the genes were mainly enriched in pathways such as small molecule catabolic process, and monosaccharide metabolic process (Fig. 2b). Similarly, KEGG pathway analysis revealed significant enrichment in carbon metabolism, glycolysis/gluconeogenesis, and tryptophan metabolism (Fig. 2c).

### Selection of hub genes and construction of the prognostic model

**Figure 3:**
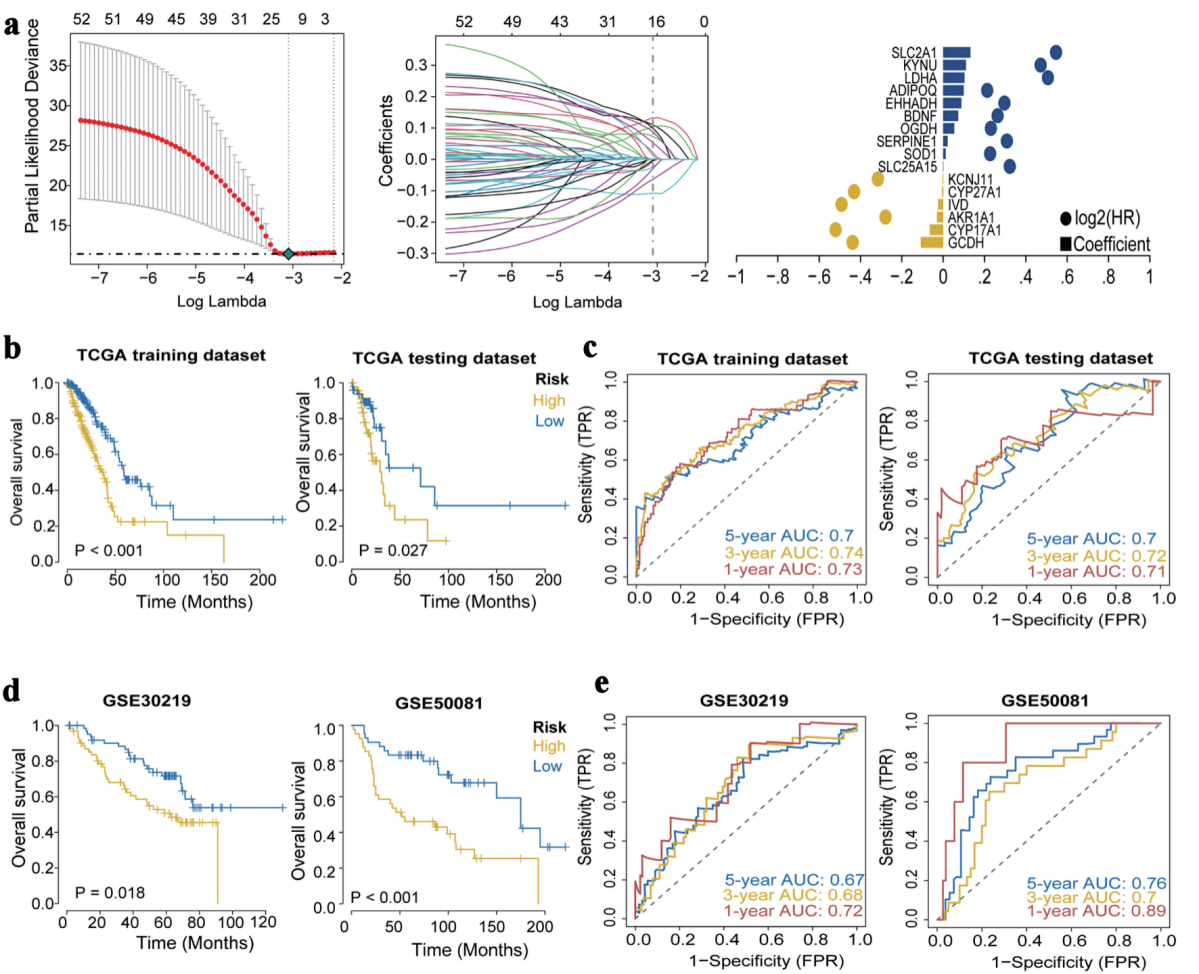
Construction and validation of the prognostic model. (a) The LASSO regression identified 16 hub genes in LUAD. (b) Kaplan-Meier survival analysis plot (P < 0.05). (c) ROC curves predicted by the risk score model for 1-year, 3-year, and 5-year OS. (d) Kaplan-Meier survival analysis plot for GEO validation. (e) Survival ROC for GEO validation. LASSO: least absolute shrinkage and selection operator; ROC: receiver operating characteristic; OS: overall survival; GEO: Gene Expression Omnibus; TCGA: The Cancer Genome Atlas; AUC: area under the receiver operating characteristic curve.

The LASSO regression feature selection algorithm based on 55 genes identified 16 hub genes (Fig. 3a). For each patient, a risk score was generated using the 16-gene formula, with coefficients derived from the LASSO model (risk score = GCDH × (-0.108260016) + CYP17A1 × (-0.063709208) + AKR1A1 × (-0.029737338) + IVD × (-0.024072127) + CYP27A1 × (-0.00551908) + KCNJ11 × (-0.003122641) + SLC25A15 × 0.000963751 + SOD1 × 0.011976998 + SERPINE1 × 0.021417855 + OGDH × 0.053367265 + BDNF × 0.072875439 + EHHADH × 0.087891887 + ADIPOQ × 0.099472524 + LDHA × 0.102977572 + KYNU × 0.110361494 + SLC2A1 × 0.13203023). Patients were then stratified into high- and low-risk groups using the median risk score as the cutoff. Kaplan-Meier results of the training set and test set showed that the overall survival (OS) of high-risk group had significantly worse OS than the low-risk group (Fig. 3b). ROC curve analysis in the training and validation cohorts further confirmed the strong predictive performance of the model (Fig. 3c).

### External dataset validation of the prognostic model’s robustness

Kaplan-Meier analyses in GSE30219 and GSE50081 demonstrated that patients in the high-risk group had significantly poorer OS than those in the low-risk group (Fig. 3d). The ROC curve results of GSE30219 and GSE50081 showed that the model had good predictive accuracy for patient prognosis (Fig. 3e).

### Association between prognosis model and immune microenvironment

**Figure 4:**
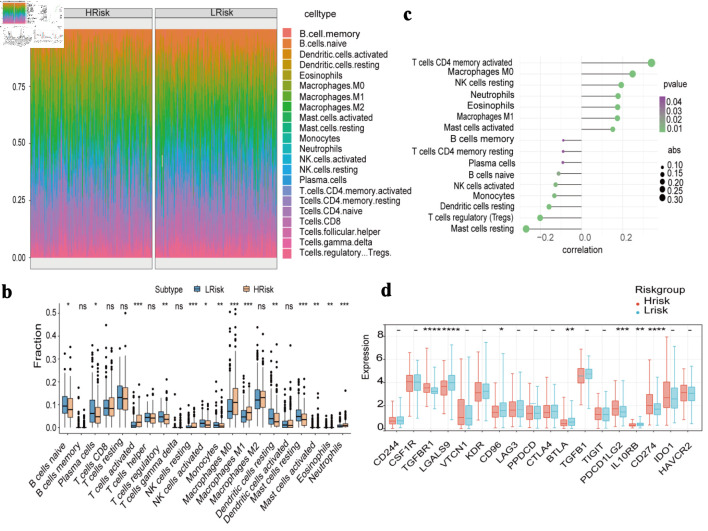
Immune cell infiltration and immune-related gene expression between high-risk and low-risk groups. (a) Heat map of the proportion of immune cells. (b) Boxplot of the proportion of immune cells. (c) Correlation analysis of immune cell content and risk score. (d) Expression differences of immune-inhibitory factors.

The relative abundance of infiltrating immune cells in the high- and low-risk groups is presented in Figure 4a. In the high-risk group, B cells naive, plasma cells, regulatory T cells (Tregs) were significantly decreased, whereas activated CD4^+^ memory T cells, M0 macrophages, M1 macrophages were significantly increased (Fig. 4b). The risk score was positively correlated with activated CD4^+^ memory T cells, M0 macrophages and negatively correlated with mast cells resting and Tregs (P < 0.05) (Fig. 4c). Differential expression analyses of immune-related chemokines, immune checkpoints, immune activators, and immune receptors between the two risk groups were also performed and visualized (Fig. 4d, Supplementary Material 2, wjon.elmerpub.com).

### Drug sensitivity and specific signaling pathway analysis

**Figure 5:**
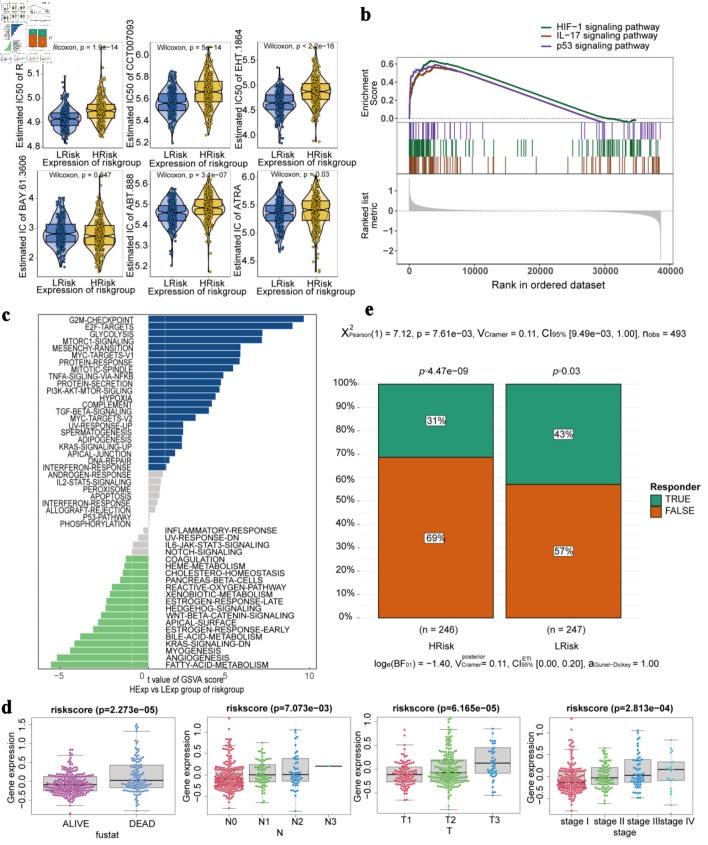
Drug sensitivity, specific signaling pathway analysis and multiple clinical indicators in high-risk and low-risk groups. (a) Drug sensitivity analysis. (b) Gene set enrichment analysis. (c) Gene set variation analysis (blue bars (*t* > 1) indicate pathways more activated in the high-risk group; green bars (*t* < -1) indicate pathways more activated in the low-risk group); the two white dotted lines represent the thresholds of *t* > 1 and *t* < -1, which were used to define biologically meaningful differences in pathway activity). (d) The difference of risk score in fustat, stage, N and T (fustat refers to “survival status,” N to “lymph node,” T to “tumor,” stage refers to “clinical staging”). (e) Immunotherapy response.

Using the R package pRRophetic, we predicted the drug sensitivity of each tumor sample and examined its association with the risk score. The results showed that the risk score was significantly associated with the predicted sensitivity to several agents, including roscovitine, CCT007093, EHT.1864, BAY.61.3606, ABT.888, and ATRA (Fig. 5a). GSEA demonstrated that the pathways enriched in the high- and low-risk groups included the hypoxia-inducible factor-1 (HIF-1), interleukin (IL)-17, and p53 signaling pathways (Fig. 5b). GSVA results for high- and low-risk groups showed that the differential pathway included G2/M checkpoint, E2F targets, and glycolysis (Fig. 5c). The gene associated groups corresponding to the three signaling pathways are shown here (Supplementary Material 3, wjon.elmerpub.com). Among these genes, *SERPINE1* is involved in HIF-1 and p53 signaling pathways.

### Correlation analysis between risk of disease and multiple clinical indicators

The Kruskal-Wallis test showed significant differences in risk score distributions according to fustat status and TNM clinical variables (stage, N, and T) (P < 0.05) Fig. 5d). Immunotherapy response also varied significantly between high- and low-risk groups (χ^2^ test, P = 7.61 × 10^-3^). Within-group response patterns were unevenly distributed (P < 0.05), with a notably higher proportion of non-responders in the high-risk group (Fig. 5e).

### Construction of nomogram and independent prognosis analysis

**Figure 6:**
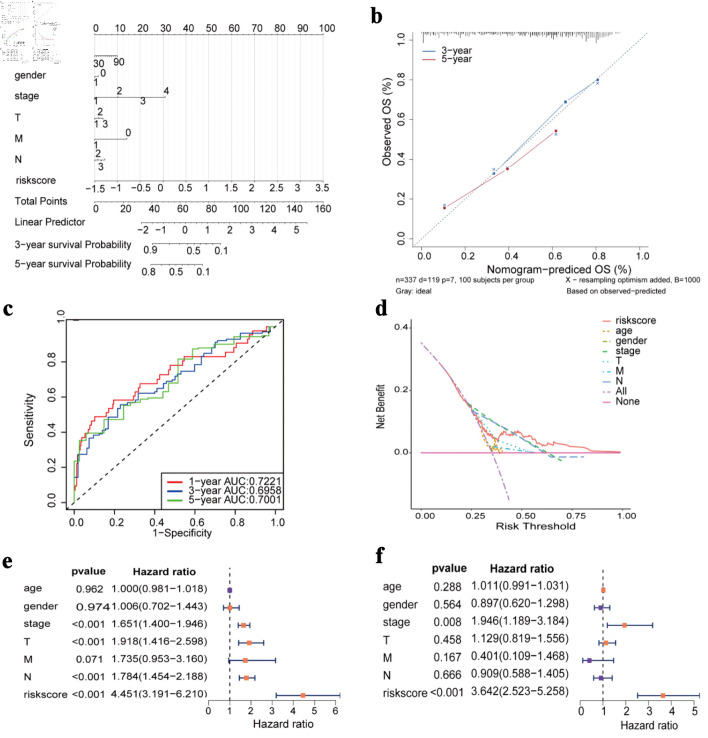
Construction of nomogram and Cox regression analysis. (a) Nomogram prediction model diagram (each variable corresponds to a point; the total points map to the survival probability). (b) Calibrated curves for 3- and 5-year specific survival. (c) ROC curves of the nomogram prediction model. (d) Decision curve analysis for nomogram. (e) Forest plot of univariate Cox regression analysis. (f) Forest plot of multivariate Cox regression analysis. OS: overall survival; AUC: area under the receiver operating characteristic curve; ROC: receiver operating characteristic.

Cox regression analysis demonstrated that the risk score was a significant predictor and contributed substantially to the construction of the nomogram (Fig. 6a). The predictive performance for 3-year and 5-year OS was evaluated, demonstrating stable and reliable discrimination across time points (Fig. 6b-d). Both univariate and multivariate Cox regression analyses confirmed that the risk score served as an independent prognostic factor for patients with LUAD (Fig. 6e, f).

### Transcriptional regulation analysis of hub genes

**Figure 7:**
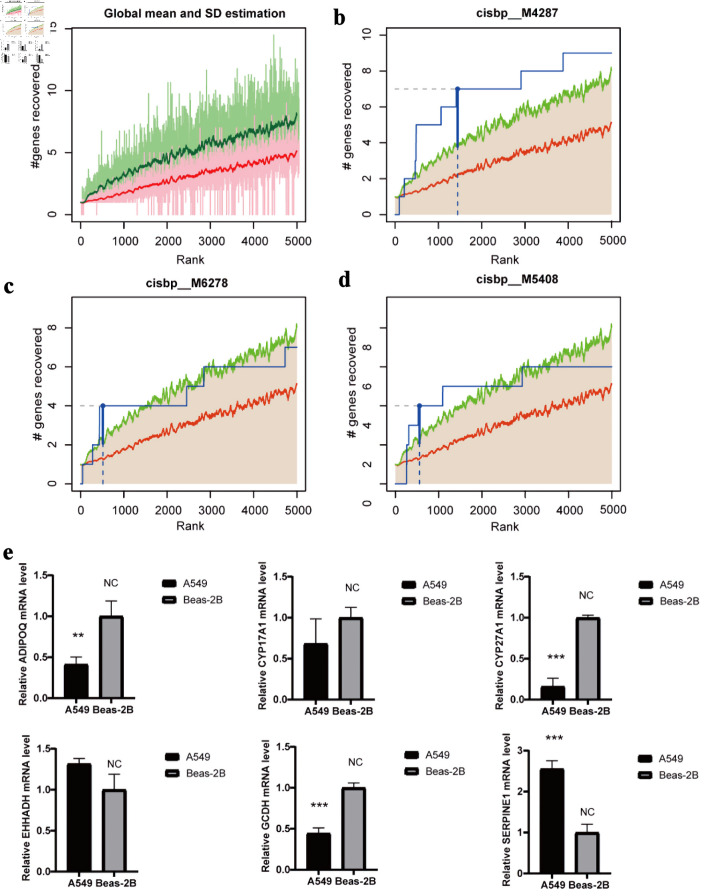
Regulatory network analysis of hub genes. (a) The enrichment analysis of transcription factors of 16 hub gene by “RcisTarget” from R package. (b) The motif of cisbp_M4287. (c) The motif of cisbp_M6278. (d) The motif of cisbp_M5408. (e) The gene expression of cisbp_M4287 in LUAD and normal epithelial cells by qRT-PCR (*P < 0.05, **P < 0.01, *** P < 0.001). LUAD: lung adenocarcinoma; qRT-PCR: quantitative real-time polymerase chain reaction.

Transcriptional regulatory analysis of the 16 hub genes was performed using cumulative recovery curves (Fig. 7a). The results showed that the most significantly enriched TF binding motif was cisbp_M4287, with a normalized enrichment score (NES) of 5.9 (Fig. 7b). Two additional motifs, cisbp_M6278 (NES = 4.77) and cisbp_M5048 (NES = 4.53), also showed notable enrichment (Fig. 7c, d). Notably, the motif with the highest enrichment score (NES: 5.9) is cisbp_M4287. Six hub genes were enriched in this motif: *ADIPOQ*, *CYP17A1*, *CYP27A1*, *EHHADH*, *GCDH*, and *SERPINE1*.

### Molecular validation experiment

qRT-PCR was performed using the LUAD cell line (A549) and the normal human bronchial epithelial cell line (Beas-2B). ADIPOQ, CYP27A1, and GCDH were significantly downregulated in A549 cells, whereas SERPINE1 showed marked upregulation compared with Beas-2B cells. The differential expression of CYP17A1 and EHHADH did not reach statistical significance (Fig. 7e).

### GWAS analysis and single-cell analysis

**Figure 8:**
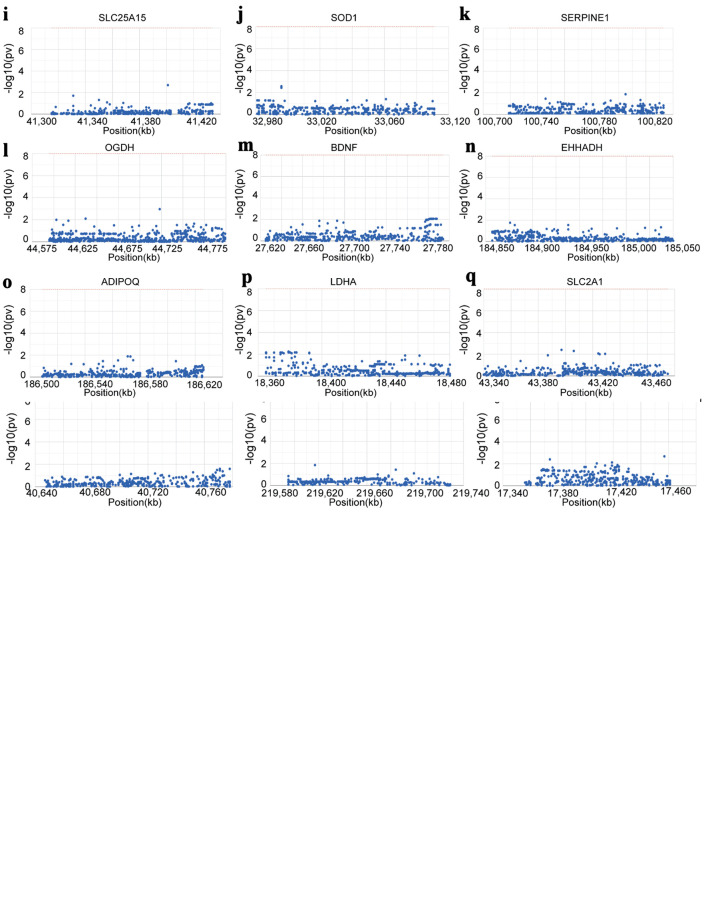
Hub genes identified by GWAS in LUAD. (a) Q-Q plot showing SNPs associated with LUAD identified by GWAS data. (b) Precise mapping of key SNP loci distributed within the enrichment regions. (c-q) Locations of the SNP-pathogenic region on the chromosome of 15 hub genes. GWAS: genome-wide association study; LUAD: lung adenocarcinoma; SNP: single nucleotide polymorphism.

We analyzed LUAD-related GWAS datasets to determine whether the hub genes harbor germline variants with potential regulatory relevance. The Q-Q plot illustrates the distribution of single nucleotide polymorphisms (SNPs) in the GWAS dataset and highlights those showing significant deviation from the expected null distribution (Fig. 8a). By precisely locating the significant SNPs in the GWAS data, we characterized the key SNP loci distributed in the genomic enrichment regions (Fig. 8b). We ultimately identified SNPs associated with 15 of the hub genes and visualized their corresponding genomic regions. Among them, *GCDH* was located in the pathogenic region of chromosome 19, *CYP17A1* in chromosome 10, *AKR1A1* in chromosome 1, *IVD* in chromosome 15, *CYP27A1* in chromosome 2, *KCNJ11* in chromosome 11, *SLC25A15* in chromosome 13, *SOD1* in chromosome 21, *SERPINE1* in chromosome 7, *OGDH* in chromosome 7, *BDNF* in chromosome 11, *EHHADH* in chromosome 3, *ADIPOQ* in chromosome 3, *LDHA* in chromosome 11, and *SLC2A1* in chromosome 1 (Fig. 8c-q).

### Single-cell analysis

**Figure 9:**
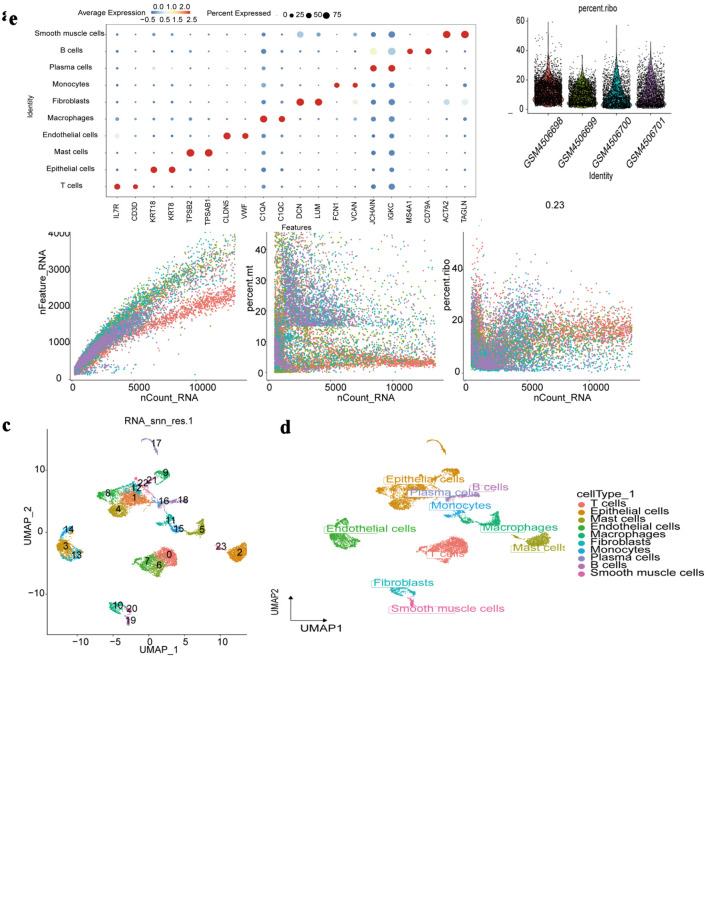
Single-cell RNA-seq profiling. (a) Distribution of quality control metrics (nCount_RNA: total RNA counts per cell; nFeature_RNA: total genes detected per cell; percent.mt: mitochondrial gene percentage; percent.ribo: ribosomal gene percentage) across individual samples (GSM identifiers). (b) Correlations between nCount_RNA and nFeature_RNA (left), percent.mt (middle), or percent.ribo (right) in all cells. Pearson correlation coefficients are labeled. (c) UMAP visualization of cell clustering (23 clusters numbered 0 - 22) based on transcriptomic profiles. (d) UMAP projection annotated by major cell types (T cells, epithelial cells, etc.) assigned via marker gene expression. (e) Dot plot showing average expression (color intensity) and percentage of cells expressing (dot size) canonical marker genes (e.g., IL7R for T cells, ACTA2 for smooth muscle cells) across each identified cell type. RNA-seq: RNA sequencing; UMAP: Uniform Manifold Approximation and Projection; IL7R: interleukin 7 receptor; ACTA2: actin alpha 2, smooth muscle.

To ensure high-quality single-cell transcriptomic data, we first performed rigorous QC by integrating metrics across all samples. Cells with outlier characteristics or fewer than 200 detected genes were removed, followed by doublet exclusion, ultimately retaining 10,053 high-quality cells for analysis. Violin and scatter plots were generated to display QC metrics (Fig. 9a, b), and 2,000 highly variable genes were identified for downstream processing. Standard preprocessing steps - including normalization, scaling, principal component analysis (PCA), and batch correction using Harmony - were subsequently applied (Supplementary Material 4A-D, wjon.elmerpub.com).

Dimensionality reduction using UMAP revealed 24 distinct cellular clusters (Fig. 9c), which were annotated into 10 major cell types based on canonical markers: T cells, epithelial cells, mast cells, endothelial cells, macrophages, fibroblasts, monocytes, plasma cells, B cells, and smooth muscle cells (Fig. 9d). Marker gene expression patterns were visualized using dot plots (Fig. 9e), and the distribution of these cell types across patient samples was summarized in bar charts (Supplementary Material 4E, wjon.elmerpub.com).

We next assessed the expression patterns of the model-related genes using DotPlot and FeaturePlot (Supplementary Material 5, wjon.elmerpub.com). ADIPOQ was not detected in the scRNA-seq dataset and was therefore excluded from downstream analyses. To distinguish malignant from non-malignant epithelial cells, we used the CopyKAT algorithm, which identified 499 aneuploid (malignant) and 2,791 diploid (non-malignant) epithelial cells.

**Figure 10:**
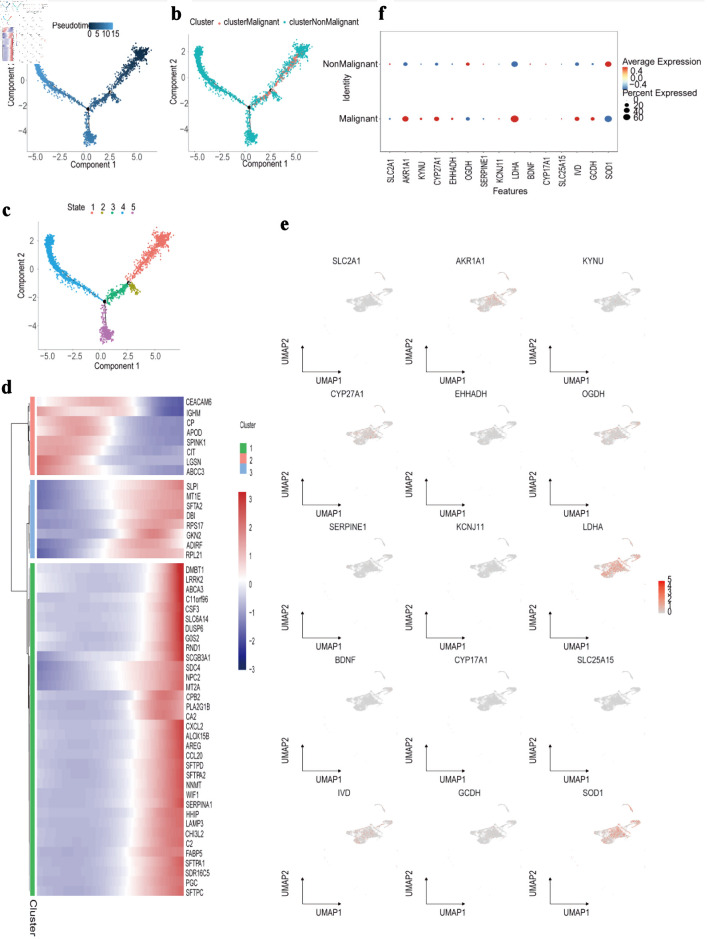
Trajectory and molecular profiling of malignant vs. non-malignant cell populations. (a) Diffusion map showing pseudotime trajectory (color gradient) of cells along components 1 and 2. (b) Diffusion map annotated by cluster (colors) and classification (malignant/non-malignant; point shape). (c) Diffusion map labeling five cellular states (colors 1 - 5) along the pseudotime trajectory. (d) Heatmap of top marker genes (rows) across cell clusters (columns), with expression scaled by row (color bar: blue = low, red = high). (e) UMAP plots showing expression distribution (color gradient: gray = low, red = high) of representative marker genes (e.g., *SLC2A1*, *LDHA*) across all cells. (f) Dot plot comparing average expression (color intensity) and percent of cells expressing (dot size) candidate genes between malignant and non-malignant cell populations. UMAP: Uniform Manifold Approximation and Projection.

**Figure 11:**
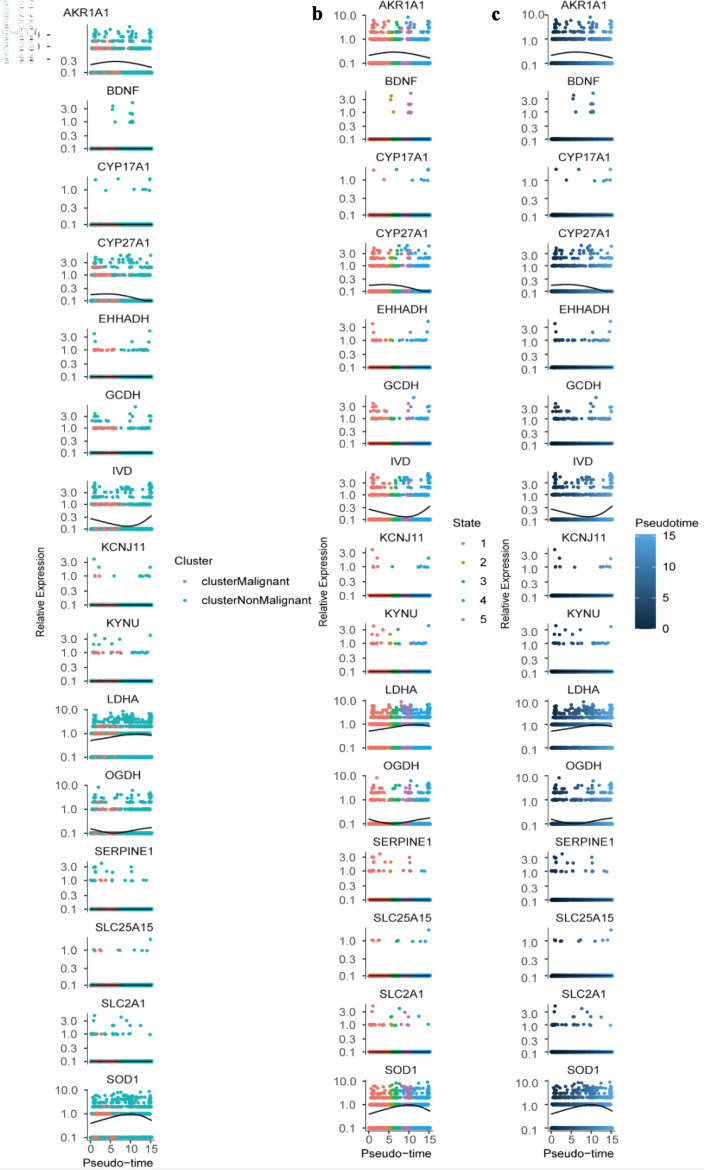
Dynamic expression of hub genes across pseudotime and cellular states. (a) Colored by cluster identity (clusterMalignant: pink; clusterNonMalignant: teal), with lines representing trend curves for each cluster. (b) Colored by cellular state (1 - 5; distinct colors), with lines indicating state-specific expression trends. (c) Colored by pseudotime (blue gradient; darker = higher pseudotime), with lines showing overall expression trajectories. All plots use a log-scaled y-axis (0.1 - 10.0) to visualize relative expression levels.

Pseudotime trajectory analysis was then conducted using Monocle to reconstruct the developmental progression of both malignant and non-malignant epithelial cells. This approach generated a detailed differentiation landscape, mapping pseudotime values, cell-state branches, and trajectory-based groupings (Fig. 10a-c). Differential pseudotime-associated genes revealed stage-specific patterns, with CEACAM6 and IGHM enriched at early differentiation stages, while DMBT1 and LRRK2 were predominantly expressed during late differentiation (Fig. 10d). Several PMRG-related genes - including *AKR1A1*, *CYP27A1*, *LDHA*, and *SOD1* - showed an early increase followed by a decline along pseudotime, whereas *IVD* and *OGDH* displayed the opposite trend (Fig. 11). Finally, the spatial distribution and expression intensity of hub genes within epithelial cell populations were visualized using DotPlot and FeaturePlot (Fig. 10e, f).

## Discussion

Currently, lung cancer is the leading cause of cancer-related deaths globally [28]. Although substantial progress has been achieved in lung cancer treatment, therapeutic outcomes remain suboptimal and continue to compromise patients’ survival and overall well-being. To address this issue, we developed a PMRG-based prognostic model using integrative bioinformatics analyses, which may facilitate personalized treatment planning and clinical decision-making in lung cancer.

The tumor microenvironment (TME), consisting of stromal and immune cells such as fibroblasts, endothelial cells, and various immune cell subsets, plays a crucial role in regulating tumor progression and therapeutic response [29]. We used the CIBERSORT algorithm to evaluate the relationship between risk scores and tumor immune infiltration. Significant differences were observed in the immune cell composition between high- and low-risk groups, particularly in naive B cells, activated CD4 memory T cells, and M0 macrophages. These distinct immune-infiltration patterns highlight the potential of our model for predicting immunotherapeutic responses. Moreover, the risk score was significantly correlated with multiple immune cell types (P < 0.05). Therefore, the risk score may serve as a reliable indicator for evaluating immune responses in patients with LUAD.

Based on the GDSC database to predict chemotherapy drug sensitivity, we discovered that certain medications might exhibit reduced efficacy in high-risk patients. This finding has implications for developing personalized treatment plans. Differential pathway analysis between high-risk and low-risk groups revealed significant enrichment of the G2/M checkpoint pathways and E2F target pathways. These two critical pathways play essential roles in tumor cell proliferation [30] and cell cycle regulation [31]. The enrichment analysis of both the high- and low-risk groups also identified the HIF-1 signaling pathway, IL-17 signaling pathway, and p53 signaling pathway, which are closely related to key biological processes such as hypoxia adaptation [32], inflammatory response [33], and DNA damage [34] during tumor development. SERPINE1 is involved in both the HIF-1 signaling pathway and the p53 signaling pathway. Hypoxia induces upregulation of SERPINE1/PAI-1 expression [35]. High levels of PAI-1 inhibit the p53 pathway and promote tumor growth [36]. These signaling pathways are closely associated with tumor onset and progression. These results further support the notion that the risk score represents a valuable prognostic indicator for LUAD.

Finally, we investigated the transcriptional regulatory mechanisms governing the hub genes. Subsequently, the motif with the highest enrichment score was validated using qRT-PCR. We found that elevated SERPINE1 expression was associated with poor prognosis. SERPINE1 inhibits plasminogen activation and reduces extracellular matrix (ECM) degradation, thereby facilitating tumor metastasis and colonization [37]. It also induces EMT by regulating TGF-β/Smad and PI3K/AKT pathways, thereby enhancing tumor cell migration and invasion [37, 38]. Therefore, SERPINE1 may serve as a potential prognostic marker and therapeutic target of tumors. We also observed that CYP17A1 and EHHADH expression did not show statistically significant differences between LUAD and normal epithelial cells. It is important to note that not all genes within a multigene prognostic model necessarily exhibit marked individual expression changes. Some may instead act as modulators or interactors within the propionate-related metabolic network. For example, propionates enhance HIF-1α expression and transcriptional activity [39], thereby influencing downstream targets (e.g., LDHA, SLC2A1, SERPINE1) and reshaping cellular metabolic programs.

This study has several limitations. First, all analyses were derived from publicly available transcriptomic datasets without matched drug-response information, preventing direct validation in immunotherapy or chemotherapy cohorts. Second, our experimental validation was preliminary; the mechanistic roles of the model genes and the functional interpretation of GWAS signals remain insufficiently explored. Third, the single-cell analysis focused mainly on expression patterns, lacking deeper functional or interaction-level investigations. Future work will include multi-center clinical cohorts with treatment response data, comprehensive mechanistic studies using cellular and animal models, multi-omics integration such as proteomics/metabolomics and organoid-based propionate perturbation assays, and in-depth expression quantitative trait locus (eQTL) and functional-variant analyses to better elucidate the biological and clinical relevance of these genes in LUAD.

### Conclusions

In conclusion, we have successfully established a prognostic model for LUAD based on hub genes in propionate metabolism. Our study investigated the associations between risk scores and tumor prognosis, immune infiltration, drug sensitivity, specific signaling pathways, and various clinical features. Furthermore, for hub genes, motif enrichment, GWAS analysis and single cell analysis were performed. These findings provide not only a theoretical basis for understanding the relationship between risk scores and LUAD prognosis but also meaningful insights into disease pathogenesis and the identification of potential therapeutic targets.

## Supplementary Material

Suppl 1 Primers used for real-time PCR.

Suppl 2 Expression differences of immune factors.

Suppl 3 Circular plot depicting key genes involved in three signaling pathways (p53, HIF-1, IL-17).

Suppl 4 Single-cell RNA-seq data processing and cell composition analysis of normal vs. tumor samples.

Suppl 5 Expression patterns of hub genes across cell populations.

## Data Availability

The datasets generated and/or analyzed during the current study are available in the public databases: TCGA database (https://portal.gdc.cancer.gov/), GEO database (http://www.ncbi.nlm.nih.gov/geo) database, and GeneCards database (https://www.genecards.org/).

## Abbreviations

LUAD: lung adenocarcinoma
TCGA: The Cancer Genome Atlas
GEO: Gene Expression Omnibus
PMRGs: propionate metabolism-related genes
EMT: epithelial-mesenchymal transition
NSCLC: non-small cell lung cancer
SCLC: small cell lung cancer
GSEA: gene set enrichment analysis
GSVA: gene set variation analysis
LASSO: least absolute shrinkage and selection operator
OS: overall survival
GO: Gene Ontology
KEGG: Kyoto Encyclopedia of Genes and Genomes
ROC: receiver operating characteristic
DCA: decision curve analysis
GDSC: Genomics of Drug Sensitivity in Cancer
qRT-PCR: quantitative real-time polymerase chain reaction
NES: normalized enrichment score
TME: tumor microenvironment
GWAS: genome-wide association study
SNPs: single nucleotide polymorphisms
UMAP: Uniform Manifold Approximation and Projection
