# Supplementary material for: Identification of Genes and Construction of Prognostic Model of Lung Adenocarcinoma Based on Propionate Metabolism-Related Genes
Source: World J Oncol. 2026 Jan 4;17(2):191–208. doi: 10.14740/wjon2680 (PMC12978388; doi:10.14740/wjon2680)
Supplement: Suppl 1 — Primers used for real-time PCR. [file wjon-17-02-191-s001.docx]

**Suppl 1.** Primers used for real-time PCR

|  | **Primer sequences** |
| --- | --- |
| ADIPOQ | TGCTGGGAGCTGTTCTACTG |
|  | TACTCCGGTTTCACCGATGTC |
| CYP17A1 | GCTGCTTACCCTAGCTTATTTGT |
|  | ACCGAATAGATGGGGCCATATTT |
| CYP27A1 | GGTGCTTTACAAGGCCAAGTA |
|  | TCCCGGTGCTCCTTCCATAG |
| EHHADH | AAACTCAGACCCGGTTGAAGA |
|  | TTGCAGAGTCTACGGGATTCT |
| GCDH | CGTCCCGAGTTTGACTGGC |
|  | GATGCGAGGCATGAGTCTCT |
| SERPINE1 | ACCGCAACGTGGTTTTCTCA |
|  | TTGAATCCCATAGCTGCTTGAAT |
| β-actin | TCTCCCAAGTCCACACAGG |
|  | GGCACGAAGGCTCATCA |
| β-actin | CTCGCCTTTGCCGATCC |
