# Supplementary material for: Identification of Genes and Construction of Prognostic Model of Lung Adenocarcinoma Based on Propionate Metabolism-Related Genes
Source: World J Oncol. 2026 Jan 4;17(2):191–208. doi: 10.14740/wjon2680 (PMC12978388; doi:10.14740/wjon2680)
Supplement: Suppl 2 — Expression differences of immune factors. [file wjon-17-02-191-s002.docx]

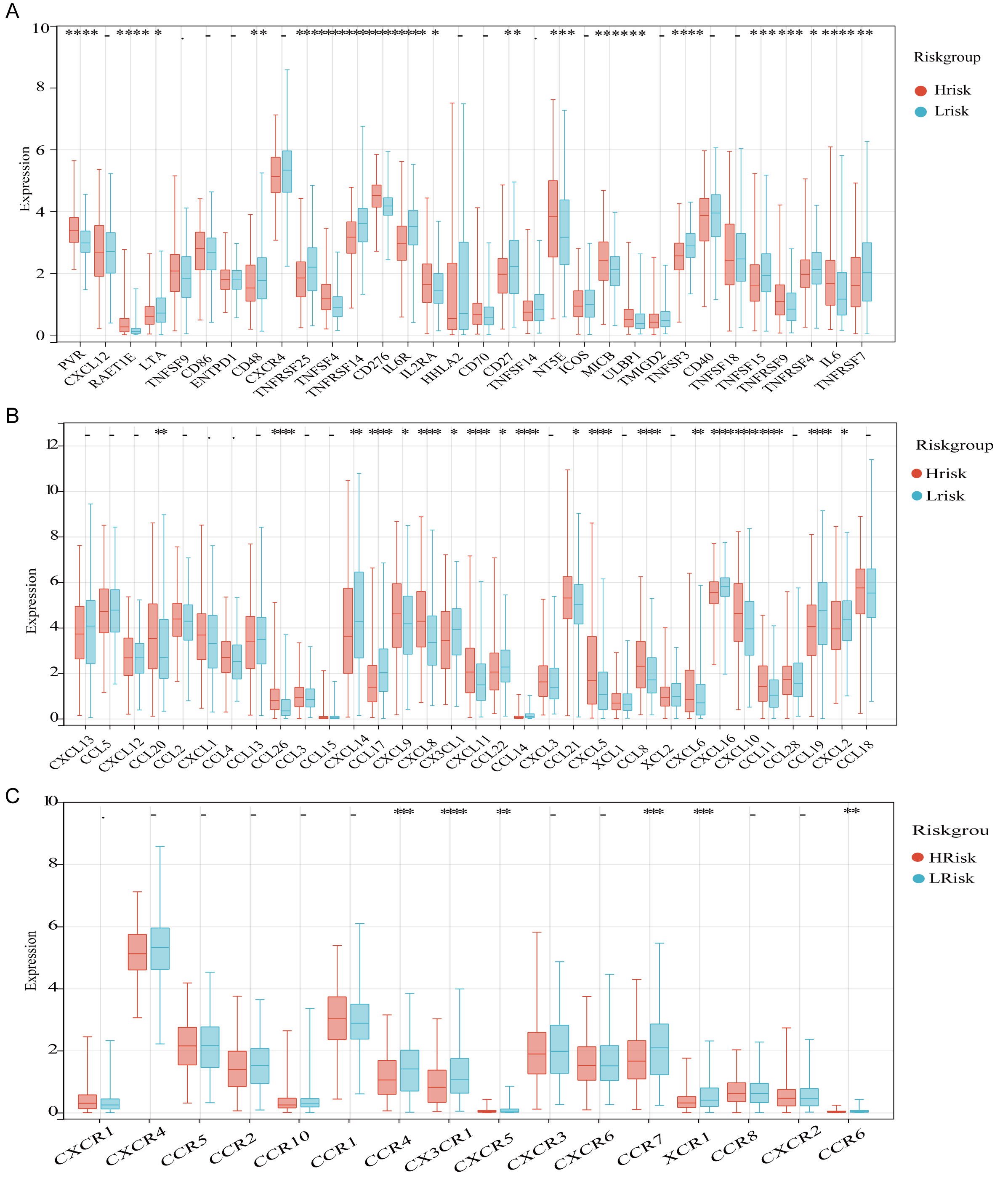


**Suppl 2.** Expression differences of immune factors. (A) Chemokines. (B) Immune-stimulatory factors. (C) Receptors.
