# Supplementary figures and images for: Identification of Genes and Construction of Prognostic Model of Lung Adenocarcinoma Based on Propionate Metabolism-Related Genes
Source: World J Oncol. 2026 Jan 4;17(2):191–208. doi: 10.14740/wjon2680 (PMC12978388; doi:10.14740/wjon2680)

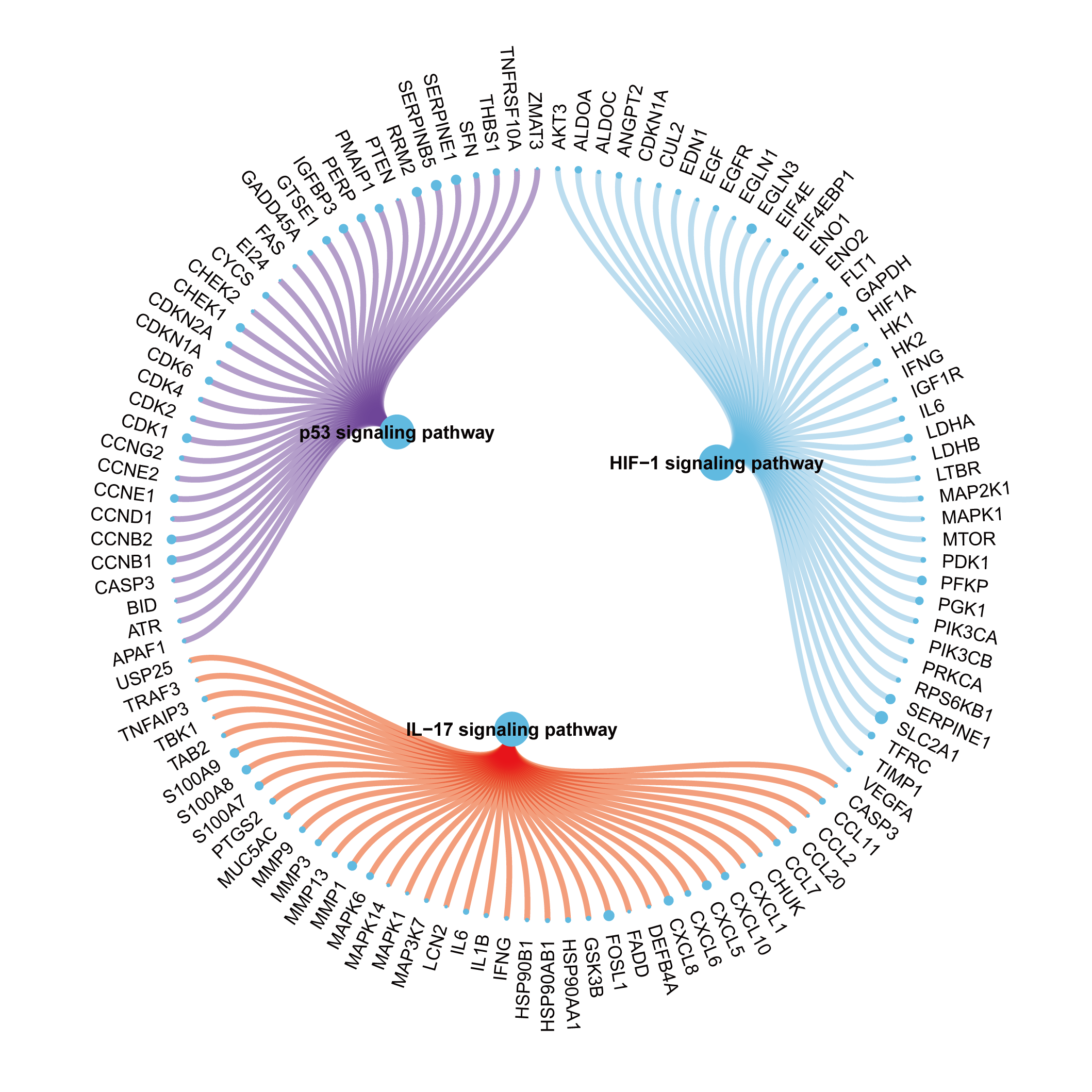


**Suppl 3.** Circular plot depicting key genes involved in three signaling pathways (p53, HIF-1, IL-17).

Supplement: Suppl 3 — Circular plot depicting key genes involved in three signaling pathways (p53, HIF-1, IL-17). [file wjon-17-02-191-s003.docx]
