# Supplementary material for: Identification of Genes and Construction of Prognostic Model of Lung Adenocarcinoma Based on Propionate Metabolism-Related Genes
Source: World J Oncol. 2026 Jan 4;17(2):191–208. doi: 10.14740/wjon2680 (PMC12978388; doi:10.14740/wjon2680)
Supplement: Suppl 4 — Single-cell RNA-seq data processing and cell composition analysis of normal vs. tumor samples. [file wjon-17-02-191-s004.docx]

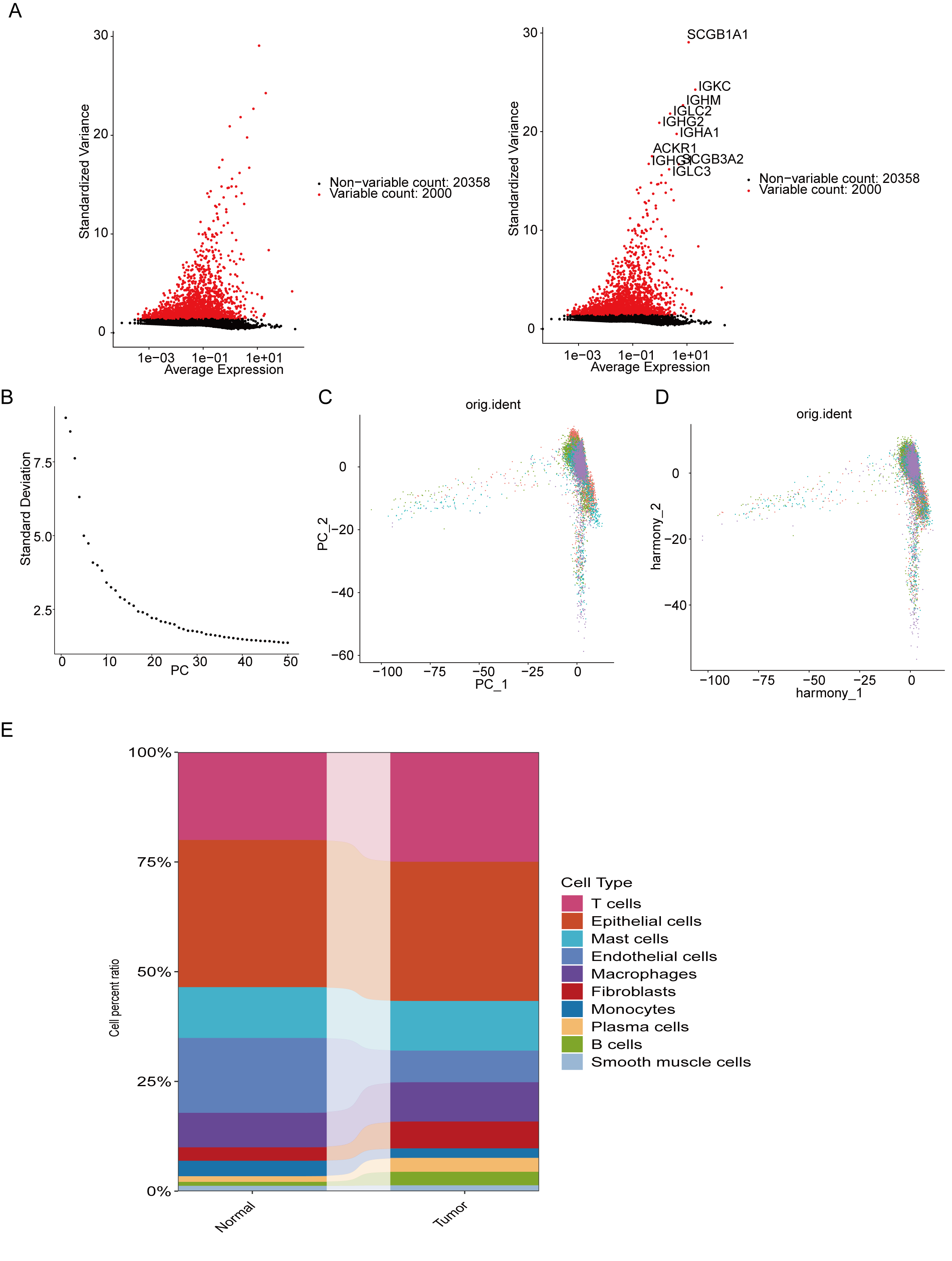


**Suppl 4.** Single-cell RNA-seq data processing and cell composition analysis of normal vs. tumor samples. (A) Gene variance plots (average expression vs. standardized variance) identifying variable genes (2,000 total, red points) and non-variable genes (20,358 total, black points); key marker genes (e.g., SCGB1A1, IGKC) are labeled in the right panel. (B) Elbow plot showing the standard deviation of principal components (PCs), used to determine the number of PCs for downstream analysis. (C) Principal component analysis (PCA). (D) Harmony-aligned embedding. (E) Stacked bar plot comparing the relative percentage of cell types (T cells, epithelial cells, etc.) between normal and tumor samples.
