# Supplementary material for: Identification of Genes and Construction of Prognostic Model of Lung Adenocarcinoma Based on Propionate Metabolism-Related Genes
Source: World J Oncol. 2026 Jan 4;17(2):191–208. doi: 10.14740/wjon2680 (PMC12978388; doi:10.14740/wjon2680)
Supplement: Suppl 5 — Expression patterns of hub genes across cell populations. [file wjon-17-02-191-s005.docx]

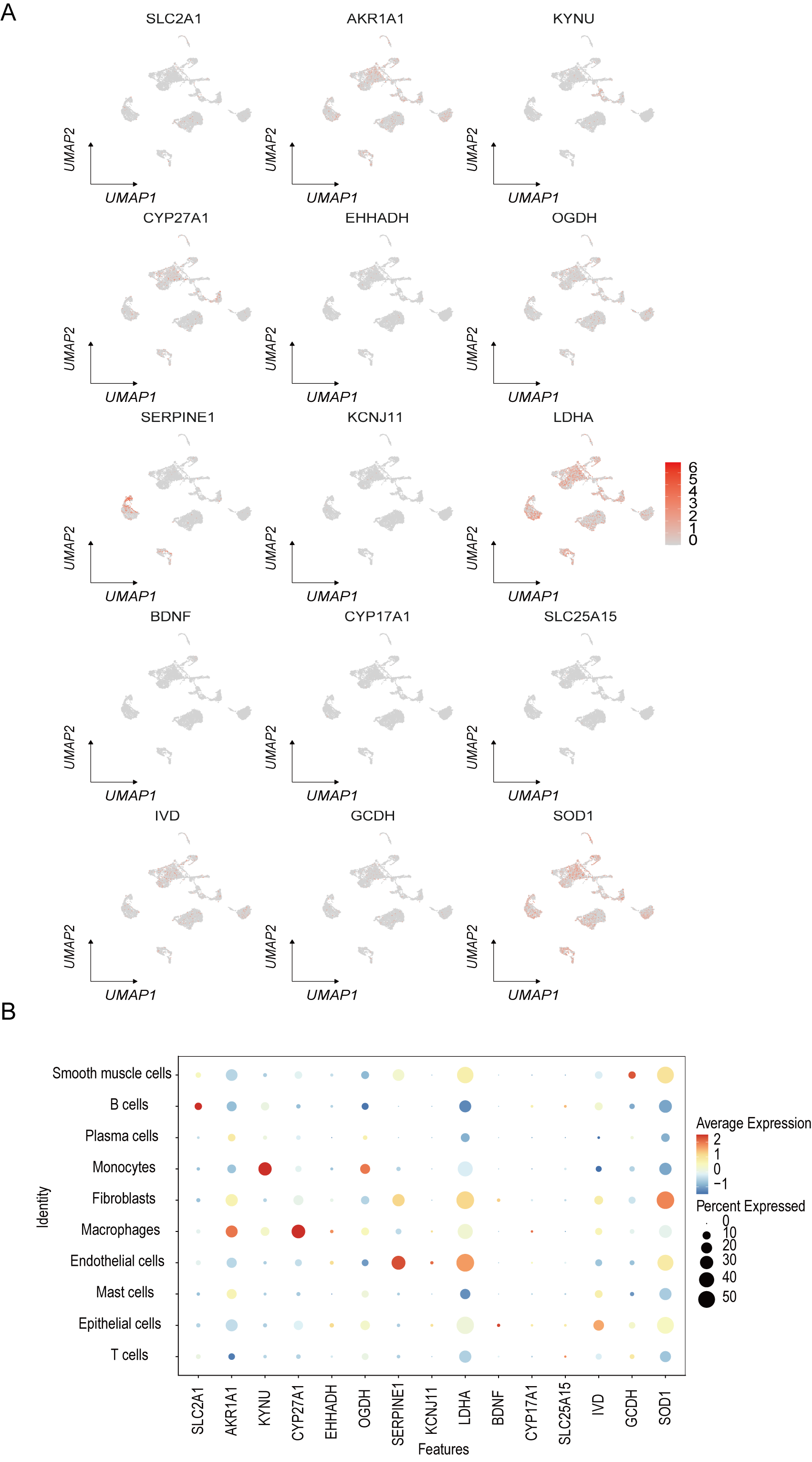


**Suppl 5.** Expression patterns of hub genes across cell populations. (A) UMAP plots showing the expression distribution (color gradient: gray = low, red = high) of 15 genes (e.g., SLC2A1, LDHA, SOD1) in single cells. Each subplot corresponds to one gene, with UMAP1/UMAP2 as axes. (B) Dot plot summarizing gene expression across cell types (rows: smooth muscle cells, B cells, etc.). Dot color indicates average expression (red = high, blue = low) and dot size indicates the percentage of cells expressing the gene (larger dots = higher percentage) within each cell type.
